# Integrated healthcare for chronically ill. Reflections on the gap between science and practice and how to bridge the gap

**DOI:** 10.5334/ijic.1079

**Published:** 2013-05-17

**Authors:** Wilma van der Vlegel-Brouwer

**Affiliations:** Policy Advisor Integrated Care, IJsselland Hospital, Pr. Constantijnweg 2, 2906 ZC, Capelle aan den IJssel, The Netherlands

**Keywords:** integrated care, chronic care, complexity, complex adaptive system, context

## Abstract

Integrated care offers an opportunity to address healthcare efficiency and effectiveness concerns and is especially relevant for elderly patients with different chronic illnesses.

In current care standards for chronic care focus is often on one disease. The chronic care model (CCM) is used as the basis of integrated care programs. It identifies essential components that encourage high-quality chronic disease care, involving the community and health system and including self-management support, delivery system design, decision support, and clinical information systems. Improvements in those interrelated components can produce system reform in which informed, activated patients interact with prepared, proactive practice teams. There is however a lack of research evidence for the impact of the chronic care model as a full model.

Integrated care programs have widely varying definitions and components and failure to recognize these variations leads to inappropriate conclusions about the effectiveness of these programs and to inappropriate application of research results. It seems important to carefully consider the type and amount of data that are collected within the disease management programs for several purposes, as well as the methods of data collection.

Understanding and changing the behavior of complex dynamic chronic care system requires an appreciation of its key patterns, leverage points and constraints. A different theoretical framework, that embraces complexity, is required. Research should be design-based, context bound and address relationships among agents in order to provide solutions that address locally defined demands and circumstances.

## Introduction

This paper reflects on current policy, research and programs of integrated care, reveals the gap between science and practice and provides a new perspective on research and development of integrated care.

Because of a higher number of elderly dependant service users with chronic illnesses and limited financial resources we seek fundamental changes in the way healthcare systems operate. Integrated care offers an opportunity to address healthcare efficiency and effectiveness concerns. This is a multi-level, multi-modal, demand driven and patient-centered strategy designed to address complex and costly health needs by achieving better coordination of services across the entire care continuum [1]. Healthcare providers inside and outside the hospital should work together to reach this goal.

The World Health Organization has defined ‘integrated care’ as ‘*the bringing together of inputs, delivery, management and organization related to diagnosis, treatment, care, rehabilitation and health promotion*’. Integrated care is a way ‘to improve services in relation to access, user satisfaction and efficiency’ [2].

However elderly and persons with chronic, disabling, medically, fragile or high risk conditions bear the brunt of access, continuity, fragmentation and quality problems found in all health systems. Current organizational structures and techniques, such as disease management and case management, are frequently confused with being integrated care [1, 3]. Many programs for chronic care are written for groups with one disease from the perspective of the professional. The perspective of the patient is often underexposed, differences and different needs of patients are not addressed in these programs and the fact that elderly patients have often more than one chronic disease is not taken into account [4–11]. Also in hospitals care is fragmented and patients with multiple illnesses carry not only the burden of their illnesses, but also the burden of their multiple treatments [4, 12].

## Dutch policy and prevention

In current healthcare the focus in the Netherlands and abroad is on health protection, health promotion, disease prevention and chronic disease management.

The World Health Organization defines health since 1948 as a state of complete physical, mental, and social well-being and not merely the absence of disease or infirmity [13]. The aim of health protection, health promotion and disease prevention is preventing diseases from occurring. In 1998, the World Health Organization, in addressing ‘disease’ prevention, stated that it ‘covers measures not only to prevent the occurrence of disease, such as risk factor reduction, but also to arrest its progress and reduce its consequences once established’ [14].

There are several definitions of prevention. Prevention, according to Caplan, is related to population groups or groups at risk. Caplan distinguishes three levels of prevention, primary, secondary and tertiary. Primary, secondary and tertiary prevention refer to different phases in the process where the problem unfolds. *Primary* prevention typically involves a broad and sweeping effort aimed at a larger group of people: at this stage it is not yet known which individuals will develop or be exposed to the problem. *Secondary* prevention consists of measures aimed at individuals singled out on account of their being at risk of developing a problem or some adverse development. *Tertiary* prevention focuses on existing, manifest problems or identified problem individuals, for instance people who have an illness or who are addicted to drugs [15]. There are two pathways to prevention arising from two fundamentally different paradigms, one medical and one behavioral. The first pathway requires the early diagnosis and treatment of disease. The second pathway promotes healthy lifestyle and disregards the requirement that a condition must be diagnosed before intervention is recommended [16].

The Dutch Government policymakes healthy living a priority [17]. Collective, primary prevention is a task for the government. It includes activities that prevent a specific health problem, illness or accident to healthy people [18]. The policy vision ‘Being healthy, staying healthy’ states that people should take care of their own health and behavior and prevention should be integrated into community care and embedded in care standards for chronic diseases [19–25]. According to the Public Health Status in the Netherlands each year 13 miljard euros are spent on prevention [26]. From this amount 80% is spent on health protection, *disease prevention 17% and health promotion 3%* [27]. Current policy is not shown by an increase on investment on prevention in the public health domain.

Health insurance for individuals covers care related secondary and tertiary prevention [28]. This care has to be evidence based or based on professional guidelines. Nearly all 135 guidelines for professionals describe how to prevent or delay the disease and how to reduce restrictions. These guidelines show a multidisciplinary approach. Guidelines, however, are not always applied in community care and hospital care and evidence about effects on clinical outcomes are varied [29, 30].

## Programmatic approach in the Netherlands and the chronic care model

The Ministry of Health, Welfare and Sports states in her policy that a programmatic approach is needed in the organization of chronic care [31]. The chronic care model (CCM) is used as the basis of disease management programs. It identifies essential components that encourage high-quality chronic disease care, involving the community and health system and including self-management support, delivery system design, decision support, and clinical information systems [32, 33]. The cronic care model is based upon a Cochrane systematic review of chronic care interventions. This review consisted of a synthesis of randomized controlled trials and controlled before and after studies of different aspects of chronic care. Taken together these findings and ongoing evaluations have shaped the model [34].

Integrated care programs seem to have positive effects on the quality of care [35–37]. However, integrated care programs have widely varying definitions and components and failure to recognize these variations leads to inappropriate conclusions about the effectiveness of these programs and to inappropriate application of research results. To compare programs and better understand the (cost) effectiveness of the programes, consistent definitions must be used and component interventions must be well described [38]. A variety of programs and interventions can be labeled disease management. Not all types of disease management programs are receiving the same level of policy interest, nor are all types equally well researched. In addition, evaluations commonly have design flaws, limiting the validity of their conclusions [39]. The standard models used in research of complex public health interventions are inadequate. They adopt a simple empiricist theoretical foundation and attempt to graft onto an essentially open social system a contrived laboratory experimentation typically in the form of a randomized, controlled trial [40].

Current models, programs and research on integrated care are fragmented and have been generally linked to specific diagnoses and indicators and do not take different levels of patients needs into consideration. According to Leutz not every patient needs fully integrated care. Depending on the severity of the chronic illness(es) linkage, coordination or full integration of care is required. Leutz divided service users into three groups: those with mild-to-moderate but stable conditions, a need for a select few routine care services and with high capacity for self-direction or strong informal networks; those with moderate levels of need; and those with long-term, severe, unstable conditions who frequently require urgent intervention from various sectors and who have limited capacity for self-direction [4].

The current disease management programs for by instant for people with diabetes or COPD show a large diversity in care programs and have not been implemented in every area in the Netherlands [41, 42]. It seems that the groups that could benefit most from a programmatic approach, the frail elderly with multi morbidity, chronically ill with limitations and the chronically ill with less health skills get the least support when dealing with their illness [43]. Findings show that practitioners do not follow established practice guidelines and there is a lack of coordination and of active follow-up to ensure the best outcomes [9, 14, 41, 44]. Attention for prevention is limited. The outcomes show what healthcare providers wish to measure and do not show improved health outcomes. Patients are not sufficiently involved in their own disease management program and patients are inadequately trained to manage their illnesses [45].

The Netherlands Organization for Health and Development, ZonMw, funds health research and stimulates use of the knowledge developed to help improve health and healthcare in the Netherlands. Vrijhoef analyzed 104 current research projects of ZonMW on integrated care [46]. Projects have a great variety of target groups and health issues, but mostly address chronic care. A majority of the projects have more than one type of integration, but full integration is hardly available. Looking at the chronic care model projects pay attention to several components, but health systems and community get little attention. Complex changes still need to be made, considering patients perspective, financial, normative and systematic integration of care. Most of the projects have a randomized controlled trial as a research design. This means more attention for process and outcome and less attention for structure. There is hardly any systematic data collection. Characteristics of integrated care as a framework for evaluation of the impact on all aspects of the quality of care are poorly applied. To support policy-makers more research is needed on the question which patients need what level of integrated care and what impact this care has on structure, process and outcome and the relationship between these variables.

Vrijhoef recommends to use of the building blocks of the chronic care model in an integrated fashion and aimed at the needs of patients, to evaluate care transformations not in isolation and to use adequate performance indicators [9]. The board of Health and Care confirms this point of view in two reports [24, 47].

*There is however a lack of research evidence for the impact of the chronic care model as a full model in ‘real world chaotic practice’* [34].

## International research

In many European countries an overview of existing disease management programs, their features and outcomes is lacking [45]. Research shows that interventions in European countries generally focus on specific diseases rather than determinants and are often insufficiently coordinated [2]. No statistically significant reductions in health service utilization are found [48].

In an international review of healthcare in the USA, UK, Netherlands, Sweden, Canada, New Zealand and Australia it is acknowledged that the chronic care model as a useful conceptual framework which provides for understanding some of the elements considered essential for the management of chronic disease and the interplay between the elements. The elements that most frequently impacted on physiological measures of disease, health and function status, and quality of life were self-management support and delivery system design particularly when in combination. Decision support and clinical information systems played an important role in health professional adherence to guidelines. There is a lack of literature for the impact of interventions focused on two elements of the model—Health Care Organization and Community Resources. These elements are relatively difficult to assess experimentally but in the real world may be of considerable importance to the overall success of chronic disease management programs. This review states that the chronic care model, while a very helpful conceptual framework, may not provide sufficient practical guidance at the level of the health service to assist policy- and decision-makers to plan and guide organization and delivery of services [49]. Gately suggests that future health policy assumptions about utilization in the context of chronic disease management and self-care support policing may benefit by acknowledging the complex, contextual and recursive nature of health service utilization operating in the real world of patients’ experience of living with a long-term condition [48].

## Future research

Complexity science offers this theoretical framework. It is the latest generation of systems theory. Complexity can be expressed as the amount of information needed to describe or understand something. An important part of complexity science is the complex adaptive system (CAS). The term ‘complex’ emphasizes that the necessary competence to perform a task is not owned by any one part, but comes as a result of co-operation within the system. ‘Adaptive’ means that system change occurs through successive adaptations [50]. Healthcare and social services can be looked upon as CAS. A CAS consists of several subsystems called ‘agents’, which are interdependent and affect each other. Agents in a CAS often have their own mental models, norms and values and assumptions. The interaction between the components leads to new behaviors and characteristics of the system. The level of connectedness between the agents defines the complexity of the network and the level of development.

In research the network has to be examined as a unified whole, considering the important role of organizational context [51, 52]. Because mental models, norms and values of agents differ locally, general solutions often do not apply. Research should be context bound and address relationships among agents in order to provide solutions that address locally defined demands and circumstances.

When human practices are the object of research practice oriented research is required. We need a different kind of knowledge production that meets the criterion of utility and studies objects in its context [53]. Design-based research is needed in order to develop knowledge which can be used by professionals in the field to design solutions to their field problems [54].

## Conclusion

Understanding and changing the behavior of the complex dynamic chronic care system requires an appreciation of its key patterns, leverage points and constraints [34].

The chronic care model is a helpful conceptual framework but does not provide sufficient practical guidance at the level of the health service to assist policy- and decision-makers to plan and guide organization and delivery of services [49]. Future health policy assumptions about utilization in the context of chronic disease management and self-care support polices may benefit by acknowledging the complex, contextual and recursive nature of health service utilization operating in the life worlds of patients’ experience of living with a long-term condition [48]. In order to develop creative and innovative strategies for the management of health care organizations research should address chronic care in its complex environment as a full model, considering all the buildings blocks of the chronic care model, including health systems and community. Because the real world is chaotic and task performance is a result of co-operation within the system future research has to address healthcare from the theory of complex adaptive systems in order to invent a full model for integrated care aimed at the needs of patients and to evaluate integrated care from a framework of characteristics on all aspects of the quality of care.

## From the author

Wilma van der Vlegel-Brouwer is a policy advisor on integrated care at the IJsselland Hospital in Capelle aan den IJssel, The Netherlands. She has been interested in integrated care for the elderly as a nurse for several years, especially since her graduation as a Master Care Trajectory Designer, the Master Degree for BA for care and welfare, University of Applied Sciences Utrecht. Her current design research focuses on integrated care and services for the frail elderly starting at the emergency department of the IJsselland Hospital.
